# Estimating longitudinal depressive symptoms from smartphone data in a transdiagnostic cohort

**DOI:** 10.1002/brb3.2077

**Published:** 2022-01-25

**Authors:** Amelia M. Pellegrini, Emily J. Huang, Patrick C. Staples, Kamber L. Hart, Jeanette M. Lorme, Hannah E. Brown, Roy H. Perlis, Jukka‐Pekka J. Onnela

**Affiliations:** ^1^ Center for Quantitative Health Massachusetts General Hospital Boston MA USA; ^2^ Department of Mathematics and Statistics Wake Forest University Winston‐Salem NC USA; ^3^ Department of Psychiatry Boston Medical Center Boston MA USA; ^4^ Department of Biostatistics Harvard T.H. Chan School of Public Health Boston MA USA

**Keywords:** bipolar disorder, ecological momentary assessment, major depressive disorder, mobile applications, schizophrenia, self report, smartphone

## Abstract

**Background:**

Passive measures collected using smartphones have been suggested to represent efficient proxies for depression severity, but the performance of such measures across diagnoses has not been studied.

**Methods:**

We enrolled a cohort of 45 individuals (11 with major depressive disorder, 11 with bipolar disorder, 11 with schizophrenia or schizoaffective disorder, and 12 individuals with no axis I psychiatric disorder). During the 8‐week study period, participants were evaluated with a rater‐administered Montgomery–Åsberg Depression Rating Scale (MADRS) biweekly, completed self‐report PHQ‐8 measures weekly on their smartphone, and consented to collection of smartphone‐based GPS and accelerometer data in order to learn about their behaviors. We utilized linear mixed models to predict depression severity on the basis of phone‐based PHQ‐8 and passive measures.

**Results:**

Among the 45 individuals, 38 (84%) completed the 8‐week study. The average root‐mean‐squared error (RMSE) in predicting the MADRS score (scale 0–60) was 4.72 using passive data alone, 4.27 using self‐report measures alone, and 4.30 using both.

**Conclusions:**

While passive measures did not improve MADRS score prediction in our cross‐disorder study, they may capture behavioral phenotypes that cannot be measured objectively, granularly, or over long‐term via self‐report.

## Graphical abstract

In this manuscript, we describe the results of an 8‐week study of a mobile application that collects a range of passive measures, investigated in 38 individuals with major depressive disorder, bipolar disorder, or schizophrenia, or screened healthy controls. Participants also underwent structured interview and clinician‐rated scales every 2 weeks.While mobile applications have been very widely discussed in psychiatry, we believe our work fills a critical need. First, it describes an open‐source, easily accessed tool that other clinical researchers can readily adopt, not a closed‐source product. Second, it realistically assesses this tool in a trial‐like design and compares it directly to clinician‐ and self‐report measures. Finally, it illustrates the range and richness of passive data, and its capacity to supplement, not replace, clinical assessment.

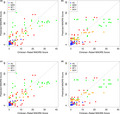

## 1 INTRODUCTION

In modern psychopharmacology, the gold standard for measurement of depressive symptoms, as with most psychiatric outcomes, has been clinician‐rated scales. However, reliance on such measures introduces substantial limitations: the need for trained clinician raters increases the cost of assessment (despite enthusiasm for measurement‐based care and recognition of the importance of quantifying treatment outcomes); the time required for clinical evaluation has precluded widespread use in practice; and clinician ratings contain sources of variance that are unrelated to underlying clinical affect. These scales themselves have been criticized for measuring a narrow range of symptoms that may be overly weighted toward specific illness features, neglecting the multidimensional nature of psychopathology (Insel et al., 2010).

The ubiquity of smartphones presents an opportunity to measure different social, cognitive, and behavioral markers in naturalistic settings. As of February 2018, 95% of Americans own a cellphone of some kind, with 77% owning a smartphone, up from just 35% in 2011 (Mobile Fact Sheet, 2018). With 6.3 billion smartphone subscriptions expected globally by 2021 (Cerwall, 2016), this technology offers an unprecedented opportunity to objectively measure human behavior in naturalistic settings outside of research laboratories and clinics.

We have previously defined the concept of digital phenotyping as the “moment‐by‐moment quantification of the individual‐level human phenotype in situ using data from personal digital devices, in particular smartphones” (Onnela & Rauch, 2016; Torous et al., 2015). Others have defined similar concepts (Glenn & Monteith, 2014; Jain et al., 2015; Monteith et al., 2015), and there is a small but growing number of studies in mental health using smartphone data (Alvarez‐Lozano et al., 2014; Benson et al., 2011; Faurholt‐Jepsen et al., 2015; Gruenerbl et al., 2014; Miskelly, 2005; Saeb et al., 2015; Torous et al., 2015; Wang et al., 2016) and other electronic devices (De Choudhury et al., 2013; Dickerson et al., 2011; Gulbahce et al., 2012; Jashinsky et al., 2014; Kane et al., 2013; Kappeler‐Setz et al., 2013; Katikalapudi et al., 2012; Matic et al., 2012; McIntyre et al., 2009; Minassian et al., 2010; Roh et al., 2012). Smartphones are well‐suited as an instrument for digital phenotyping given their widespread adoption, the extent to which users engage with the devices, and the richness of their data. Being able to accomplish this without the expense and burden associated with additional specialized equipment makes the approach attractive to researchers (Onnela & Rauch, 2016). Smartphone‐based digital phenotyping encompasses the collection of a range of different social and behavioral data, including but not limited to spatial trajectories (via GPS), physical mobility patterns (via accelerometer), social networks and communication dynamics (via call and text logs), and voice samples (via microphone) (Onnela & Rauch, 2016; Torous et al., 2015).

The performance of digital phenotyping has rarely been directly compared to clinical rating scales in trial‐like settings, nor has it been examined in a transdiagnostic cohort. To address these gaps, we conducted an 8‐week study among psychiatric outpatients with mood and psychotic disorders, as well as healthy controls. Our aim was to assess whether digital phenotyping may be used as a complement for in‐person psychiatric assessments of depressive symptoms, using the MADRS, in a clinical population. We sought to assess to what extent it might be possible to predict a future clinician‐rated score on the Montgomery–Åsberg Depression Rating Scale (MADRS) from baseline assessments of MADRS, surveys administered on the phone (here, Patient Health Questionnaire), passively collected smartphone data (here, GPS and accelerometer), or a combination of these measures. More generally, we sought to quantify data completeness, a critical but commonly overlooked question in digital phenotyping, examining GPS, accelerometer, and phone survey data over the course of the 8‐week study.

## 2 MATERIALS AND METHODS

### 2.1 Study design and cohort description

This study used a prospective cohort design and aimed to recruit equal‐sized groups of outpatients with major depressive disorder (*n* = 11), bipolar I or II disorder (*n* = 11), schizophrenia or schizoaffective disorder (*n* = 11), and screened healthy controls with no axis I psychiatric disorder (*n* = 12). Each participant's primary diagnosis was confirmed by the Structured Clinical Interview for DSM‐IV (SCID) Modules A‐D (*Diagnostic & statistical manual of mental disorders: DSM‐IV*, 2000). Demographic features of the study cohort are shown in Table 1.

**TABLE 1:** Description of cohort (*n* = 41)

Baseline Covariate	
Sex	
Male	37% (15/41)
Female	63% (26/41)
Age (years)	
Mean (*SD*)	43 (12)
Min, Q1, Q2, Q3, Max	21, 33, 45, 52, 68
Diagnosis	
Healthy control	27% (11/41)
Major depressive disorder	24% (10/41)
Bipolar disorder	24% (10/41)
Schizophrenia/schizoaffective	24% (10/41)
Race	
White	71% (29/41)
African‐American	20% (8/41)
Asian	7% (3/41)
Other	2% (1/41)
Baseline MADRS Score (mean (*SD*))	
Healthy control	0.7 (1.2)
Major depressive disorder	20.0 (12.7)
Bipolar disorder	9.7 (10.6)
Schizophrenia/schizoaffective	6.2 (5.4)

Abbreviations: MADRS, Montgomery–Åsberg Depression Rating Scale; Q1, Q2, and Q3 represent the first, second, and third quartiles; sd, standard deviation.

Participants were recruited from outpatient clinics of the Massachusetts General Hospital (Boston, MA) and via advertisements seeking healthy control participants between 2015 and 2018. All participants signed written informed consent prior to participation. The study protocol was reviewed and approved by the Partners HealthCare Institutional Review Board (protocol #: 2015P000666). Participants were compensated $50 after the initial baseline visit and an additional $100 upon completion of the study. If a participant withdrew from the study before completing the full 8 weeks, they were compensated $25 in addition to the initial $50. Participants received reimbursement for reasonable parking and travel expenses for each in‐person study visit.

All participants were 18 years or older and owned a smartphone running an iOS or Android operating system and were judged likely able to comply with study procedures by the site investigator's estimation. Participants installed the Beiwe application at the baseline visit and provided demographic information. Participants then returned for four follow‐up visits over the course of the 8 weeks (for a total of five in‐person visits, scheduled approximately every two weeks).

### 2.2 Longitudinal assessments

At baseline and each follow‐up visit, trained raters (AMP and KLH) certified and supervised by psychiatric clinical trialists (HEB and RHP) administered the MADRS. The overall MADRS score ranged from 0 to 60, and the following cutoff points were usually applied: 0 to 6 (not depressed), 7 to 19 (mild depression), 20 to 34 (moderate depression), and above 34 (severe depression). Participants also responded daily to a 4‐question in‐app Likert scale survey on overall mood, social interest, sleep quality, and activity level (Table S1). They were also prompted once a week on Saturdays to take an in‐app Patient Health Questionnaire (PHQ‐8) survey (Kroenke et al., 2009). The question assessing suicidality included in PHQ‐9 was omitted because the Partners HealthCare IRB has previously determined that its inclusion would require real‐time evaluation of patient data, deemed by the investigators to be infeasible in the present design. To remain enrolled in the study, participants were required to respond to the surveys at least five times a week.

### 2.3 Beiwe research platform

In this study, we used the Beiwe application for data collection, which is the front‐end component of the Beiwe research platform. We have previously described an earlier version of the Beiwe research platform for high‐throughput smartphone‐based digital phenotyping in biomedical research use (Torous et al., 2016). The front end of Beiwe consists of smartphone apps for iOS (by Apple) and Android (by Google) devices. The back‐end system, which enables data collection and data analysis and supports study management, makes use of Amazon Web Services (AWS)‐based cloud computing infrastructure. While data collection is arguably becoming easier with developing technology, analysis of the collected data is increasingly identified as the main bottleneck in research settings (Iniesta et al., 2016; Kubota et al., 2016; Kuehn, 2016). For this reason, Beiwe consists of a growing suite of data analysis and modeling tools triggered by the Beiwe data analysis pipeline.

Reproducibility remains a challenge in the biomedical sciences, as fewer than 10% of studies have been found fully reproducible (Prinz et al., 2011). To enhance reproducibility, all Beiwe data collection settings for both active (smartphone surveys and audio samples) and passive (smartphone sensors and logs) data are captured in a single JSON‐formatted configuration file, which can be imported to future studies to enable them to use identical data collection. The configuration for this present study is also available.

### 2.4 Data collection, storage, and security

Each study participant was assigned a randomly generated 8‐character Beiwe User ID and a temporary password, and study staff assisted participants with app installation and activation at the time of enrollment. Data collected by the Beiwe application were immediately encrypted and stored on the smartphone until the phone was connected to Wi‐Fi, at which point the data were uploaded to the study server and expunged from the phone. The reason for configuring Beiwe to use Wi‐Fi rather than cellular data in this study was to avoid charges associated with uploading large volumes of data, roughly 1GB per subject‐month, to the cloud. Any potentially identifying data were hashed on the mobile device, and all data were encrypted while stored on the phone awaiting upload, while in transit, and while on the server.

### 2.5 Processing of passive data: Phone gps and accelerometer data

During the time period between the baseline visit and the last follow‐up visit, accelerometer and GPS data from participants’ smartphones were collected using Beiwe. The GPS measured the phone's latitude/longitude coordinates, while the accelerometer measured its acceleration along three orthogonal axes. To preserve the battery life of the phone (mainly due to GPS) and to reduce data volume (mainly due to accelerometer), each sensor alternated between an on‐cycle and off‐cycle according to a predefined schedule (10 s on, 10 s off for the accelerometer; 2 min on, 10 min off for GPS). We selected a longer on‐period for the GPS as it required time to locate the satellites required for positioning its location, and we correspondingly selected a longer off‐period to reduce battery drain. In Supplemental Material, we describe our procedure for generating covariates for MADRS prediction from raw GPS and accelerometer data. Roughly speaking, the data were first summarized at a daily level and then the daily summaries were aggregated by type of day (weekend versus weekday). For Android users, in addition to accelerometer and GPS data, we collected anonymized communication logs, which we used to derive two summary statistics: the number of outgoing calls and the number of unique phone numbers dialed. Plots and summary statistics of the Android communication log data are presented in Results section.

### 2.6 Statistical analysis

We used linear mixed models for MADRS prediction. Linear mixed models are an extension of standard linear regression to clustered data, where the clusters here are multiple MADRS assessments over time for each subject. Importantly, linear mixed models can handle clusters of varying size due to missing data. We considered four main model specifications. Each of them included the baseline MADRS score and the demographic variables as predictors (Table 2). We included the baseline MADRS score as a predictor based on the following rationale. One would ideally like to predict MADRS scores from passive data only, but this would require a large sample size and may not even be possible. The next best approach is to predict future MADRS scores from passive data and some baseline MADRS data. We assumed this latter approach because this approach, if successful, could reduce the number of times the MADRS score needs to be evaluated, which would help economize healthcare resources. The models differed by which smartphone‐based covariates were included as additional predictors: Model A used phone‐based PHQ‐8 surveys, Model B used weekly summaries of passive smartphone data, Model C used both PHQ‐8 surveys and weekly summaries of passive smartphone data, and Model D used neither. In Models A and C, when including the phone‐based PHQ‐8 survey score as a predictor, we used the survey that was closest in time preceding the MADRS assessment in question. We chose to include the PHQ‐8 survey score as a predictor because of the ease of completion on a mobile phone by patients, and because of its widespread use as a screen in primary care settings. For Models B and C, we sought to predict MADRS score based on passive smartphone data collected in the seven days preceding the MADRS assessment. We computed summary statistics using raw GPS and accelerometer data. Our previous work has shown that one needs to impute missing GPS data when constructing summary statistics from GPS data. To generate summary statistics from GPS data, we first imputed missing GPS trajectories using a resampling method that has previously been demonstrated to result in a 10‐fold reduction in the error averaged across all mobility features compared to simple linear interpolation of data by Barnett and Onnela (2018). After imputing missing data, we then computed several GPS summaries proposed by Canzian and Musolesi (2015), Saeb et al. (2015), and Barnett and Onnela (2020). There were 32 candidate summary statistics computed from smartphone passive data (GPS and accelerometer) (see Table 2 and Table S2). As many of these statistics were correlated, rather than including all 32 statistics as predictors in the models that used passive data, we performed a principal component analysis (PCA) on the 32 summary statistics and used the first principal component as a predictor. For each model, we performed leave‐one‐subject‐out cross‐validation to evaluate its prediction accuracy. This entailed holding out the data from each participant in turn, fitting the model with the data from the other participants, and using the fixed effects portion of the fitted model to predict the MADRS scores of the held‐out participant. At the model‐fitting step, we excluded data points with missing values for one or more of the predictors. As our accuracy metric, we computed the root‐mean‐squared error (RMSE) for each participant and then took the average across all participants. To compute the RMSE for each participant, we took the squared error between the predicted and actual MADRS score for each visit, averaged the squared errors across all visits for the given participant, and finally took the square root of this quantity. A lower RMSE indicates more accurate predictions. Of note, as a preliminary investigation, we elect to present model fit rather than statistical comparisons of models.

**TABLE 2:** Predictors used in the study

Baseline predictors	Predictors based on phone surveys	Predictors based on passive smartphone data^a^
Age Sex Diagnostic category MADRS score	Score on the PHQ‐8 survey closest in time preceding the MADRS assessment	GPS‐based^b^ Number of significant locations visited Time spent at home Distance traveled Maximum diameter Maximum home distance Radius of gyration Average flight length Standard deviation of flight length Average flight duration Standard deviation of flight duration Probability of pause Significant location entropy Circadian routine Weekend–weekday routine Number of minutes with missing data Accelerometer‐based^b^ Activity level Number of minutes with missing data

Abbreviations: MADRS, Montgomery–Åsberg Depression Rating Scale; PHQ‐8, Patient Health Questionnaire‐8.

^a^ These predictors are defined in Methods S1.

^b^ Except for number of minutes with missing data, all other GPS‐based or accelerometer‐based predictors were computed separately for weekdays versus weekends.

## 3 RESULTS

### 3.1 Participant baseline covariates and MADRS scores

**FIGURE 1:**
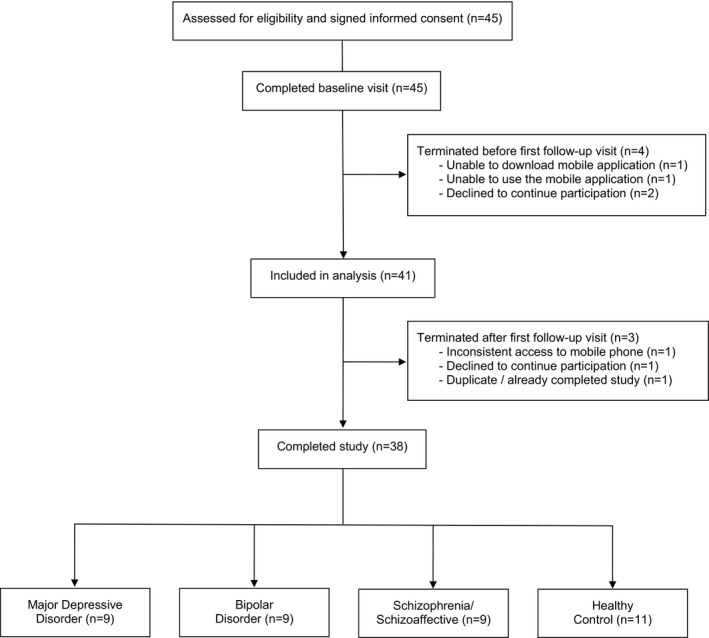
CONSORT flow diagram

Of the 45 consented participants, we excluded four participants who elected to cease study participation at or before the first follow‐up visit (Figure 1). All other participants (*n* = 41) were included in the analysis, of whom three participants dropped out after the first follow‐up visit and 38 fully completed the 8‐week study. Table 1 shows the baseline features of these 41 study participants, including age, sex, diagnostic category, race, and baseline MADRS score. There were no missing data for these features. For the participants who completed the study, MADRS scores were available at baseline and at each of the four follow‐up visits. Among the three participants who dropped out after the first follow‐up visit, MADRS scores were assessed for two participants at the first follow‐up visit. For descriptive purposes, Figure S1a, b shows the participants’ MADRS trajectories over time, and a scatterplot of the average of the MADRS scores versus the standard deviation of the MADRS scores for each subject.

### 3.2 Assessing completeness of phone data

The completeness of the accelerometer and GPS data was assessed at the participant level. For the accelerometer, we divided the number of minutes of data actually collected by the number of minutes of data expected to be collected. We examined the time period ranging from the day after the baseline visit to the day before the last follow‐up visit, i.e., the time period including all full days in the study. Since accelerometer data were scheduled to be collected every minute, the expected number of minutes with data was the number of minutes in the time period. The completeness of GPS data was assessed analogously, except the expected number of minutes of data was 1/6 of the number of minutes in the time period (the 2‐min on‐cycle is 1/6 of the total cycle). The proportions for accelerometer and GPS are shown in Figure S2a. The proportions are variable, ranging from 0 to 0.99 for accelerometer and from 0 to 0.87 for GPS. For the accelerometer, 23 out of 41 (56%) participants had proportions of 0.5 or higher. For GPS, 16 out of 41 (39%) participants had proportions of 0.5 or higher. The proportions tended to be greater for accelerometer data than for GPS data. Despite the missingness, a large amount of data was captured over the course of the study, including 674,969,086 accelerometer measurements and 14,733,731 GPS measurements. The quantity of collected data for iOS phones tended to be greater on average than for Android phones.

Figure S2b shows the completion rate for each PHQ‐8 survey, indicated by the solid black line. Given a specific survey, its completion rate was defined as the proportion of participants who completed the survey. If a participant completed Survey *t* after Survey *t + *1 had been sent, they were counted as not having completed Survey *t* but were counted as having completed Survey *t + *1. The completion rate was 95% for the first survey and 80% for the last survey, which took place approximately two months after the baseline visit. Figure S3 shows a histogram of the number of weeks that the participant completed one or more PHQ‐8 surveys. If a participant completed more than one survey during some week (i.e., the participant was late on the previous week's survey), the multiple surveys only contributed 1 to the participant's tally. Overall, 78% of participants completed PHQ‐8 surveys on 8 or more weeks.

**FIGURE 2:**
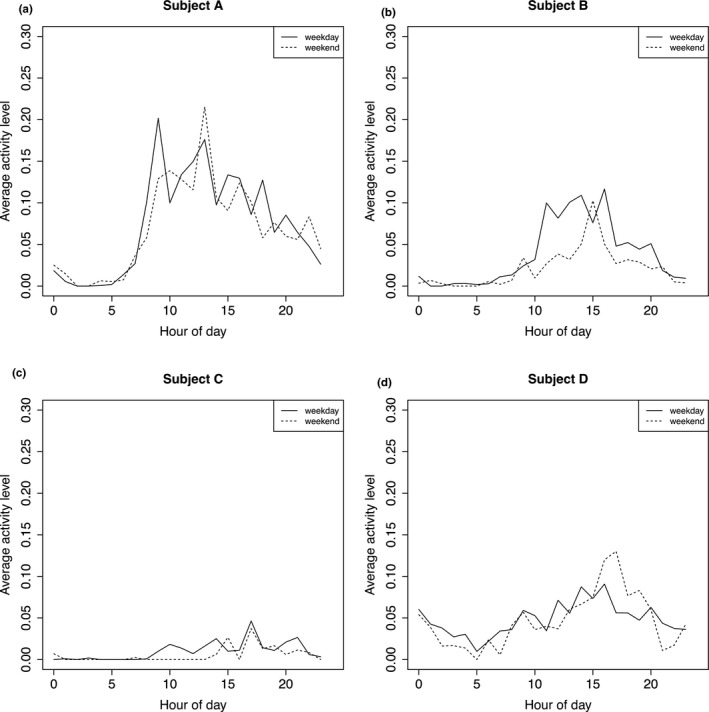
(a‐d). Average activity level from 12:00 a.m. to 11:59 p.m. on weekdays and weekends for four randomly selected participants in the schizophrenia/schizoaffective diagnostic group. The solid line corresponds to the weekday, and the dotted line to the weekend. The x‐axis origin of hour = 0 corresponds to 12:00 a.m. See Methods S1 for details on how these curves were computed

As an example of passive data, Figure 2a‐d plots the average activity level hour‐by‐hour (from 12:00 a.m. to 11:59 p.m.) for four randomly chosen participants in the schizophrenia/schizoaffective group on weekdays and weekends. For each participant, the curves were computed using accelerometer data collected throughout their follow‐up as described in detail in Supplemental Material. For any given 1‐hr window, the average activity level estimates the proportion of time that the participant was active (e.g., walking, using stairs) compared to stationary (e.g., sitting, standing, lying down) during this hour of the day. On weekdays, the participant in Panel A had low activity levels overnight, which began rising around 7 a.m., and hit their highest levels between 9 a.m. and 1 p.m., followed by a decline over the course of the evening. On weekends, their activity level was lower in the morning than on weekdays and was highest at 1 p.m. In interpreting these plots, a caveat is that the participant's activity was missed if the phone was not carried (e.g., it was left on a table). Thus, differences between the participants could be due to differences in their activity patterns, as well as differences in their phone use habits (e.g., how often each participant carried their phone).

**FIGURE 3:**
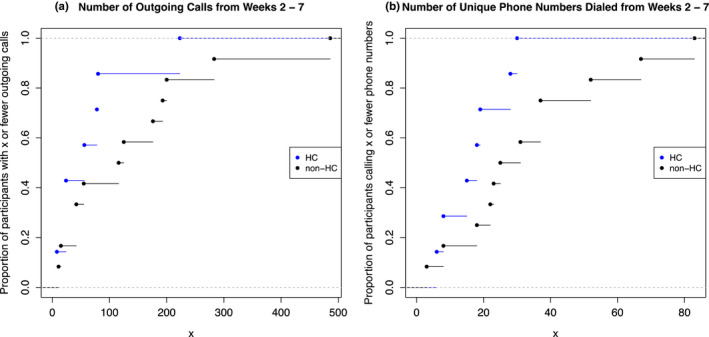
Cumulative distribution functions for the number of outgoing calls (a) and the number of dialed phone numbers (b) among Android participants, stratified by healthy control status. HC, healthy control; non‐HC, individuals with a psychiatric diagnosis

Data completeness for each passive modality, and for self‐report, is summarized in Supplemental Results. In addition, we collected smartphone communication logs from Android devices (no iOS devices were included in this part of the analysis). Figure 3 shows the cumulative distribution functions for the number of outgoing phone calls and the number of unique phone numbers dialed over Weeks 2–7, stratified by status (healthy control versus schizophrenia/schizoaffective, bipolar, or major depressive disorder). All individuals included here had communication log data collected throughout Weeks 2–7. Among this subset of participants (*n* = 19), the median age was 33 years (IQR: 29 – 41) for healthy controls (*n* = 7) and 52 years (IQR: 43–55) for others (*n* = 12). The proportions of female participants were 43% and 83%, respectively. The median number of outgoing calls was 56 (IQR: 24–79) for the healthy controls compared to 121 (IQR: 42–195) for those with a psychiatric diagnosis. The median number of unique phone numbers was also lower for the healthy controls at 18 (IQR: 12–24) versus 28 (IQR: 21–41) for those with a psychiatric diagnosis.

### 3.3 MADRS prediction

**FIGURE 4:**
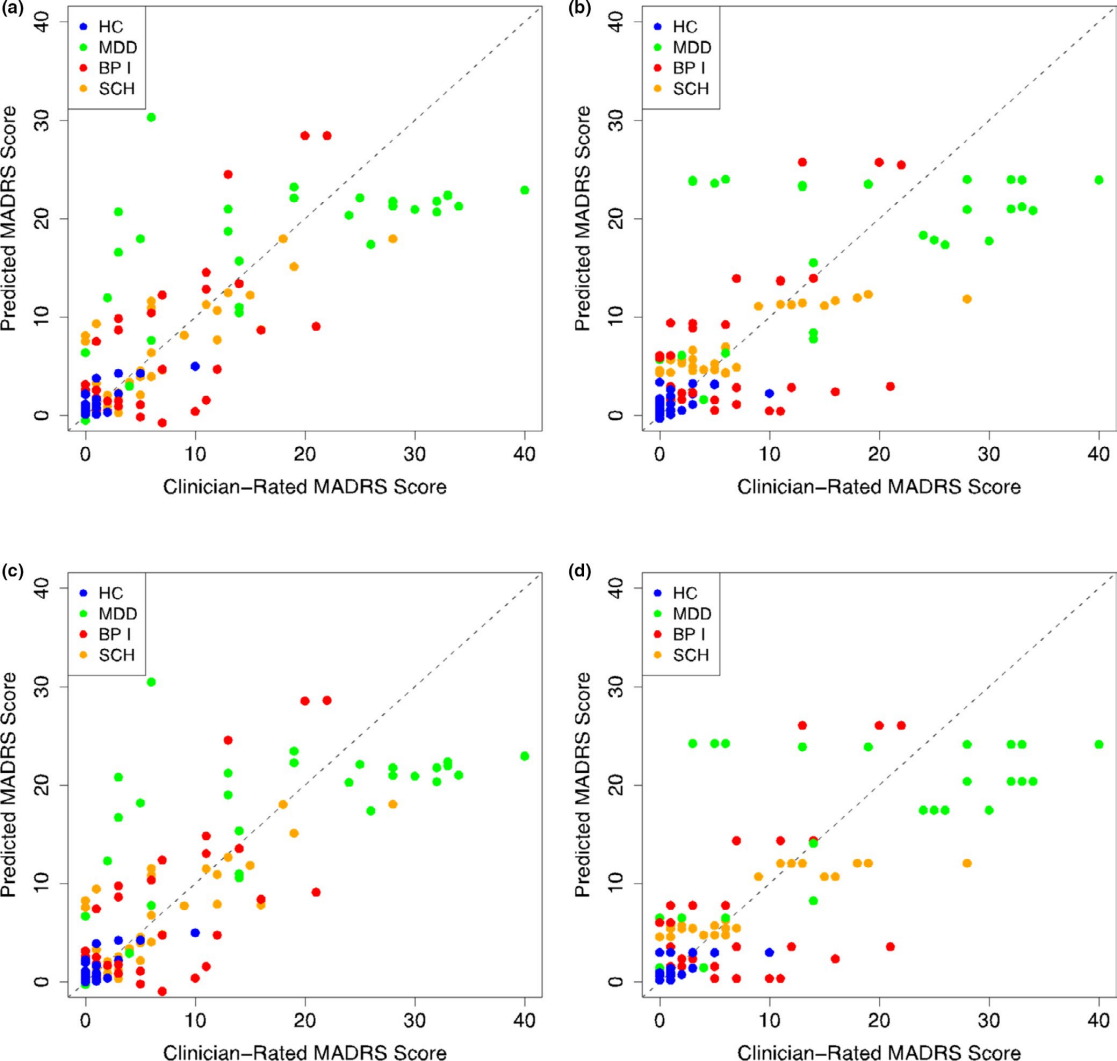
Montgomery–Åsberg Depression Rating Scale (MADRS) score predictions using four models. All models included age, sex, diagnostic category, and baseline MADRS score as predictors. They differed by which phone‐based variables were used as predictors: Patient Health Questionnaire‐8 survey scores only (a), passive data only (b), both (c), and neither (d). BP I, bipolar disorder; HC, healthy control; MDD, major depressive disorder; SCH, schizophrenia/schizoaffective disorder

Figure 4a–d shows the predicted MADRS scores compared to the clinician‐rated MADRS scores. Panels A–D correspond to Models A–D: Panel/Model A (baseline MADRS & demographics & PHQ‐8); Panel/Model B (baseline MADRS & demographics & passive data); Panel/Model C (baseline MADRS & demographics & PHQ‐8 & passive data); and Panel/Model D (baseline MADRS & demographics). For the models that included passive data, we performed a principal component analysis and used the first principal component as a predictor. When principal component analysis was applied without excluding any subjects, the first principal component explained 46% of the variance in the data and the highest weights came from GPS‐based features. In Table S3, we provide the weighting for each sensor‐based feature in the first principal component. The predicted MADRS scores were computed using leave‐one‐subject‐out cross‐validation, as described above. The average RMSE was 4.27 for Model A, 4.72 for Model B, 4.30 for Model C, and 4.66 for Model D. That is, incorporation of passive variables in Model B did not meaningfully improve the average RMSE compared to using only the baseline MADRS score and demographics in Model D.

Models A–D each included both baseline MADRS and demographics as predictors. Although basic demographic variables can be easily collected and incorporated in the model, baseline MADRS scores might not be commonly available. To assess prediction accuracy in this modified setting, we next omitted the baseline MADRS from each model and otherwise proceeded as above. The average RMSE was 5.46 for Model A’ (demographics & PHQ‐8), 6.99 for Model B’ (demographics & passive data), 5.46 for Model C’ (demographics & PHQ‐8 & passive data), and 6.91 for Model D’ (demographics). The inclusion of baseline MADRS scores improves the average RMSE by approximately 1 point if PHQ‐8 is included and 2 points if PHQ‐8 is not included. Finally, as a sensitivity analysis, if demographics are also omitted, we obtain the following RMSE values: 5.69 for Model A’’ (PHQ‐8), 7.94 for Model B’’ (passive data), 5.72 for Model C’’ (PHQ‐8 & passive data), 7.95 for Model D’’ (no predictors, only an intercept). Results for different models are summarized in Table 3.

**TABLE 3:** The average root‐mean‐squared error (RMSE) in predicting the MADRS score (scale 0–60) using three different variants of Models A–D

		+ demographics	+ baseline MADRS + demographics
Model A (PHQ−8)	5.69	5.46	4.27
Model B (passive data)	7.94	6.99	4.72
Model C (PHQ−8 & passive)	5.72	5.46	4.30
Model D (no phone‐based predictors)	7.95 (intercept only)	6.91	4.66
Model E (first and second principal component)	7.86	6.97	4.75
Model F (distance traveled on weekend)	7.96	6.97	4.72

Abbreviations: MADRS, Montgomery–Åsberg Depression Rating Scale; PHQ‐8, Patient Health Questionnaire‐8.

As an exploratory analysis, we evaluated the effect of including the second principal component (PC) as a predictor, which we call Model E. The results are shown in Table 3 in the row entitled Model E. Comparing the average RMSE’s after adding the second PC (Model E) relative to having the first PC only (Model B), the average RMSE slightly improves when there are no other variables in the model or when the other variables are demographics, but slightly worsens when baseline MADRS and demographics are included. We conducted a separate exploratory analysis in which we identified the variables that had the highest loadings in the first PC: distance traveled maximum diameter, maximum home distance, and radius of gyration on the weekend. Since it is the most interpretable of the four, we used distance traveled on the weekend as the single passive predictor in a new model called Model F, which had no PC’s included. This led to similar average RMSE’s as using the first PC (Model B) and using the first and second PC (Model E).

## 4 DISCUSSION

In this cross‐disorder investigation, we found that including passive data as a predictor did not improve the prediction of clinician‐rated MADRS scores. While the participant payment employed in this study precludes strong conclusions about acceptability, the high retention rate suggests that, with compensation, participants are willing to adopt this technology as part of a standard clinical assessment model. A similar approach has successfully been used in other settings, such as to study patients with schizophrenia, where the subjects were not paid for app use, not given additional support for app use, and not provided with check‐in calls or study staff reminders to use the app (Barnett et al., 2018).

Both academic researchers and pharmaceutical leaders have suggested that passive measures may replace clinical evaluation in clinical trials as a means of improving signal detection (Harvey et al., 2018). Setting aside the need for clinician involvement to ensure participant safety, our results suggest that more work will be required to replace clinical raters for assessment of MADRS.

Although passive data did not perform as well as phone‐based PHQ‐8 in terms of average RMSE, it is important to stress that the passive approach requires only a one‐time installation of the application which, even if less precise, may be valuable in settings where individuals are unlikely to adhere to a survey protocol, especially for extended time periods and in the absence of financial or other incentives.

One possible explanation for why incorporating passive variables in Model B did not improve the average RMSE compared to using only the baseline MADRS score and demographics in Model D is the varying data quality among participants. For example, Figure S4 shows the availability of accelerometer data for three participants. For each hour over the course of the follow‐up, we plot the proportion of minutes with accelerometer data collected. A shading of white corresponds to 0 (no data collected during that hour), black to 1 (data collected at every minute), and different shadings of gray to in‐between values. The *x*‐axis shows the week of the follow‐up, and the y‐axis shows the day of the week with the tick marks occurring at 12:00 a.m. The participant in the top panel had high data quality throughout their follow‐up. The participant in the middle panel had high data quality during most of the study with some long gaps with no data. The participant in the bottom panel had some medium data quality periods interspersed with periods with no data. Using incomplete passive data to predict the MADRS score can be challenging since the timing of the missing gaps may not be random (Figure S4). When deriving our predictors from passive data, we avoided the naïve approach of taking averages across the available data, which would overweight time intervals during which data tended to be collected. Instead, we utilized a more robust method for handling missingness, which is described in Methods S1. However, the predictors may be inaccurate when the proportions of data collected are low (Figure S2a).

In a meta‐analysis of seven smartphone‐based digital phenotyping studies, there was no significant difference found in levels of missing data by sex, age, educational background, and phone operating system for either accelerometer or GPS data (Kiang et al., 2019). Another study found that levels of missing GPS and accelerometer data were predictive of future clinical survey scores in a cohort of patients with schizophrenia (Torous et al., 2018), which presents a potential future extension of the analyses presented here.

We note multiple important limitations in considering our results. First, the study design precludes conclusions about application of smartphone apps in longer‐term studies or those using 'lighter touch' designs without in‐person visits. Second, we cannot exclude the possibility that additional passive measures, or alternate means of analyzing such measures, will yield better prediction of clinician ratings. Indeed, our work should encourage other investigators to apply our open‐source platform and further develop our analytic methodologies. Our analyses mix between‐person and within‐person variation in MADRS scores. Since these are distinct types of variation, a potential area of future research is to separately assess within‐person changes from between‐person differences. Third, because of the IRB‐mandated omission of the PHQ suicide item, we likely underestimate the ability of this measure to capture more severe depression. Fourth, as a pilot study, sample size is modest and thus the result that passive measures do not significantly contribute to predicting MADRS must be viewed as preliminary. In future studies, strategies to reduce missing data (for example, by monitoring data missingness for each participant during the course of the study and intervening where required) merit consideration. Higher data quality may help improve the utility of passive measures.

We also emphasize strengths of using passively collected smartphone data in psychiatric settings. Passive data likely capture depressive features that are not well‐measured by clinical raters, such as physical activity levels, spatial isolation (as measured via GPS‐based home time), and social isolation (as measured via communication logs). Investigation of this hypothesis represents an important priority for clinical investigators seeking to develop a next generation of pragmatic trials. In other words, rather than simply replacing clinical raters, passive measures may themselves represent useful biomarkers, but only if they can be validated for this role.

We elected to conduct a cross‐disorder study to recognize that categorical diagnosis fails to capture the dimensional nature of psychopathology, consistent with the NIMH’s Research Domain Criteria framework (Insel et al., 2010). That is, it may be useful to capture negative valence symptoms such as depression across a range of disorders, not just in major depressive disorder. While such symptoms may be attributed to different underlying processes (e.g., negative symptoms in schizophrenia), our results suggest the ability of a single platform to measure across disorders.

## 5 CONCLUSION

While passively collected smartphone data did not improve the prediction of MADRS scores in our cross‐disorder study, we demonstrate its application to capture features of patients’ daily functioning—such as physical activity, social isolation, and spatial isolation—that are otherwise difficult to capture with surveys. These various behavioral phenotypes, which are listed in Table 2 and defined in the Supplement, can describe participants' physical activity (e.g., from the accelerometer data), spatial isolation (e.g., time spent at home, computed from GPS data), and social isolation (e.g., number of outgoing calls from Android call log data).

## 6 HUMAN SUBJECTS ETHICS STATEMENT

All participants signed written informed consent prior to their inclusion in the study. The study protocol was reviewed and approved by the Partners HealthCare Institutional Review Board (protocol #: 2015P000666).

## CONFLICT OF INTEREST

AMP, EJH, KLH, and JML have no disclosures to report. PCS is employed by Mindstrong Health, Inc. HEB receives research funding from Janssen Pharmaceutica, Acadia Pharmaceuticals, and the Stanley Medical Research Institute. RHP receives research funding from NIMH, NHLBI, NHGRI, and Telefonica Alfa. RHP holds equity in Psy Therapeutics and Outermost Therapeutics; serves on the scientific advisory board of Genomind, Psy Therapeutics, and Outermost Therapeutics; consults to RID Ventures and Takeda; and receives salary support from *JAMA Network‐Open* for service as Associate Editor. JPO receives research funding from NIH, Harvard University, Otsuka Pharmaceutical, and Apple; he has also received an unrestricted gift from Mindstrong Health, Inc.

## AUTHOR CONTRIBUTIONS

All authors have contributed meaningfully to this work and gave final approval to submit for publication.

### Peer Review

The peer review history for this article is available at https://publons.com/publon/10.1002/brb3.2077.

## Supporting information

Supplementary Material

## Data Availability

IRB approval does not permit public data release in light of concerns about reidentifiability (McCoy & Hughes, 2018). Investigators seeking access to data are encouraged to contact the authors.
