# Supplementary material for: Estimating longitudinal depressive symptoms from smartphone data in a transdiagnostic cohort
Source: Brain Behav. 2022 Jan 25;12(2):e02077. doi: 10.1002/brb3.2077 (PMC8865149; doi:10.1002/brb3.2077)

**Supporting Information**

*Supplemental Methods*

To impute the missing gaps in the GPS data, we used the resampling method we have earlier developed for this purpose; compared to naïve linear interpolation, this imputation has been shown to result in a 10-fold reduction in the error averaged across all daily mobility features (e.g., daily distance traveled) used in subsequent analyses (Barnett & Onnela, 2020). Briefly, the method first turns the sequence of GPS latitude-longitude coordinates into a mobility trajectory consisting of flights (events consisting of straight-line movement) and pauses (events consisting of essentially no movement). It then fills in the gaps in the flight-pause mobility trajectory by resampling events from the joint distribution of flights and pauses by applying an appropriate resampling kernel to upweight those observations that occur either close in time (modulo 24 hours), space, or both to the missing data events. We summarized the continuous (after imputation) mobility trajectories in terms of 15 daily summary statistics, which consist of summaries such as total distance traveled and time spent at home, the location of which was inferred from data using another method (Canzian & Musolesi, 2015). These daily summary statistics are defined in Supplemental Table 2. To compute the GPS-based covariates to predict a given MADRS score, we took the average of the daily summaries in the 7 days prior to the MADRS assessment, stratified by type of day (weekend versus weekdays). This was done for all daily summaries except amount of missing data, yielding 29 GPS-based predictors. For each subject, we used accelerometer data to estimate the average weekday activity level and the average weekend activity level during the 7 days prior to the MADRS assessment. To estimate the average weekday activity level, we looked separately at four time intervals: 12:00 AM – 5:59 AM, 6:00 AM – 11:59 AM, 12:00 PM – 5:59 PM, 6:00 PM – 11:59 PM. For each time interval, we computed the total number of minutes accelerometer data was collected within the time interval on the five weekdays of the 7-day window. For each of these minutes, we computed the variance of the acceleration values separately for each of the three orthogonal axes and added the three variances together. The proportion of minutes with a variance sum exceeding 0.15 *g*^2^ was then used as an estimate of the average weekday activity level during the particular time interval. This procedure was performed for each of the four daily time intervals. The average weekday activity level was computed by averaging the four resulting values. The activity level for the weekend was computed analogously, except using the accelerometer data collected on the Saturday and Sunday during the 7-day window. We treated weekdays and weekends separately since people’s activity patterns differed between these two types of days. Also, the activity levels were estimated within time interval first and then averaged across time intervals because the amount of accelerometer data collected can vary over the course of a day; had we weighted each minute of available data equally, the activity levels would have been influenced disproportionately by the time intervals of the day that had more data available. The choice of the threshold value of 0.15 *g^2^* was made based on tests performed in our lab to identify a reasonable threshold for differentiating between movement activities (e.g., walking, ascending stairs, descending stairs) and stationary activities (e.g., standing, sitting). Although the coarse approach used here is not suitable for distinguishing between different types of physical activity, it provides an overall measure of physical activity performed while carrying the phone.

To compute the weekday average activity level curve for a given participant, we considered one hour of the day at a time. As an example, for the hour from 12:00 AM – 12:59 AM, we counted the number of minutes of accelerometer data collected during that time window on weekdays throughout the participant’s study follow-up. We then computed the proportion of the minutes with variance sum exceeding the threshold of 0.15 *g^2^*. This was taken to be the participant’s average activity level on weekdays from 12:00 AM – 12:59 AM. We performed this procedure for each hour of the day, yielding an hour-by-hour weekday activity level curve. We computed the participant’s weekend average activity level curve analogously, except only data collected on weekends were used.

*Supplemental Methods References*

Barnett, I., & Onnela, J.-P. (2020). Inferring Mobility Measures from GPS Traces with

Missing Data. *Biostatistics*, 21(2), e98-e112.

https://doi.org/10.1093/biostatistics/kxy059

Canzian, L., & Musolesi, M. (2015). Trajectories of depression: unobtrusive monitoring of depressive states by means of smartphone mobility traces analysis. *Proceedings of the 2015 ACM International Joint Conference on Pervasive and Ubiquitous Computing (UbiComp ’15)*, 1293–1304. https://doi.org/10.1145/2750858.2805845

*Supplemental Tables and Figures*

**Supplemental Table 1.** Instructions and survey questions for the in-app Likert scale survey.

| **Instructions**: Please answer the questions below on a 7-point scale. 1 = much less or worse than usual. 4 = about average. 7 = much more or better than usual.   1. How interested were you in talking, texting, or emailing with other people today? 2. How active were you today? 3. How good was your mood today? 4. How well did you sleep last night? |
| --- |

**Supplemental Table 2.** Definitions of GPS-based daily summary statistics.

| **GPS-based daily summary statistic** | **Definition** |
| --- | --- |
| Number of significant locations visited | Number of significant locations visited during the day determined based on the data from their entire follow-up using k-means clustering. |
| Time spent at home | Amount of time spent at home during the day. Home is defined as the significant location visited most frequently during the time interval of 8 PM to 8 AM, over the course of the subject’s follow-up. |
| Distance travelled | Distance travelled over the course of the day. |
| Maximum diameter | The largest distance between any two places visited during the day. |
| Maximum home distance | For each place visited during the day, the distance from home is computed. The maximum home distance is the largest of these distances. |
| Radius of gyration | Average radius that a person travels from their “center” on that day. On any given day, the person’s center is computed by taking the weighted average the *(x,y)*-coordinates for each place visited during the day, weighted by the time the person spent at the place during the day. Radius of gyration is the weighted average of the distance between each place and the center, with the same weights. |
| Average flight length | Movement is partitioned into flights and pauses. These summary statistics give the averages and standard deviations of flight length (i.e., distance) and flight duration (i.e., time). |
| Standard deviation of flight length |  |
| Average flight duration |  |
| Standard deviation of flight duration |  |
| Probability of pause | The fraction of the day spent in pause (i.e., not moving). |
| Significant location entropy | For each significant location that was visited, we compute the proportion of the day spent at the location multiplied by the natural logarithm of the proportion. The significant location entropy is the sum of these values, multiplied by -1. |
| Circadian routine | A score between 0 and 1 that assesses how closely the person follows their routine. The subject’s routine is computed using GPS data collected throughout their follow-up, treating each day equally. |
| Weekend-weekday routine | A score between 0 and 1 that assesses how closely the person followed their weekday routine that day (if the day is a weekday) or their weekend routine (if the day is on a weekend). |
| Number of minutes with missing data | Number of minutes in the day during which no GPS data was collected. |

**Supplemental Table 3.** Weighting for each GPS-based and accelerometer-based feature in the first principal component after applying principal component analysis to all subjects.

|  | **Weekend** | **Weekday** |
| --- | --- | --- |
| **GPS-based** | | |
| Number of significant locations visited | 0.08 | 0.08 |
| Time spent at home | -0.19 | -0.20 |
| Distance travelled | 0.23 | 0.22 |
| Maximum diameter | 0.23 | 0.22 |
| Maximum home distance | 0.23 | 0.21 |
| Radius of gyration | 0.23 | 0.21 |
| Average flight length | 0.21 | 0.20 |
| Standard deviation of flight length | 0.22 | 0.21 |
| Average flight duration | 0.13 | 0.10 |
| Standard deviation of flight duration | 0.16 | 0.14 |
| Probability of pause | -0.14 | -0.10 |
| Significant location entropy | 0.09 | 0.09 |
| Circadian routine | - 0.22 | -0.21 |
| Weekend-weekday routine | - 0.21 | -0.20 |
| Number of minutes with missing data | 0.08 | |
| **Accelerometer-based** | | |
| Activity level | 0.14 | 0.12 |
| Number of minutes with missing data | 0.09 | |

**Supplemental Figure 1A-B**. At the top (A), participants’ MADRS scores over time. Each trajectory corresponds to a unique participant, and the color of the trajectory indicates her/his diagnostic category. MADRS = Montgomery–Åsberg Depression Rating Scale; HC = Healthy Control; MDD = Major Depressive Disorder; BP I = Bipolar Disorder; SCH = Schizophrenia/Schizoaffective Disorder. At the bottom (B), a scatterplot showing each participant’s average MADRS score across the baseline and follow-up visits, versus the standard deviation of her/his MADRS scores.

**A.**


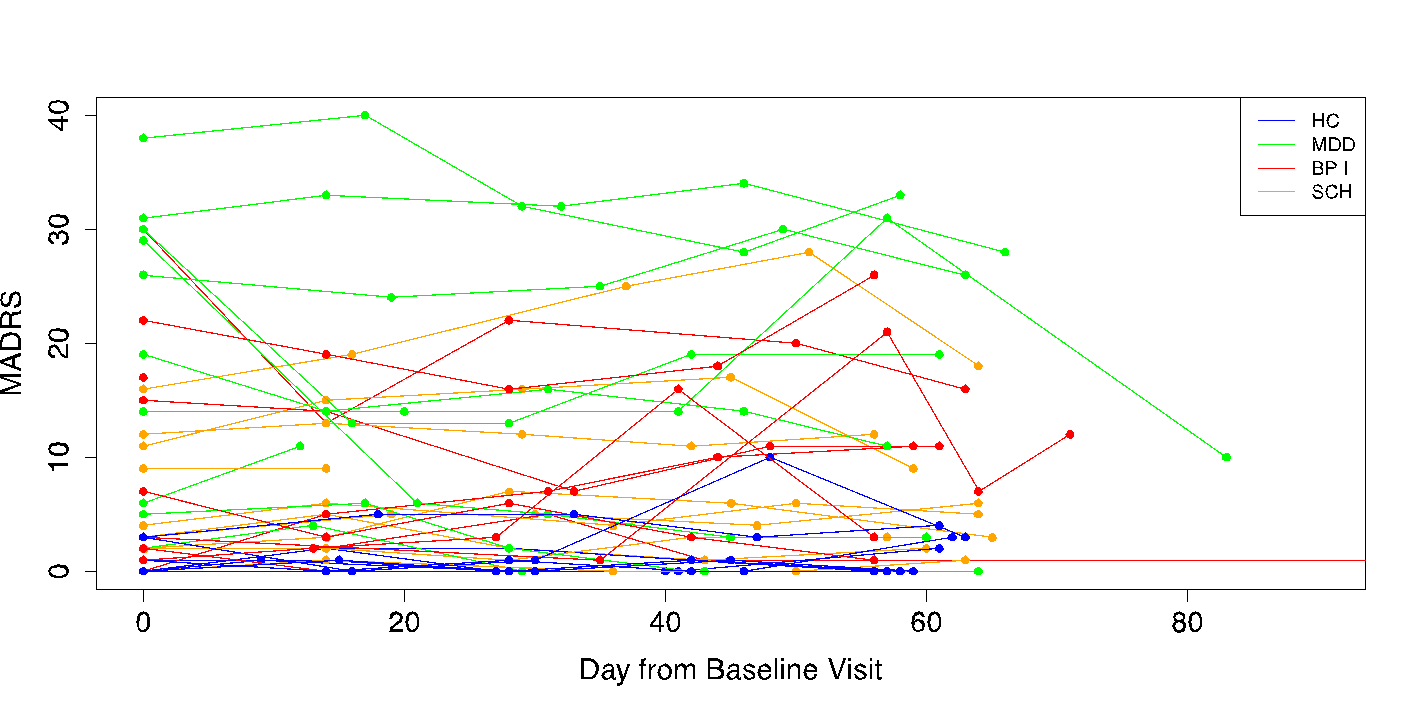


**B.
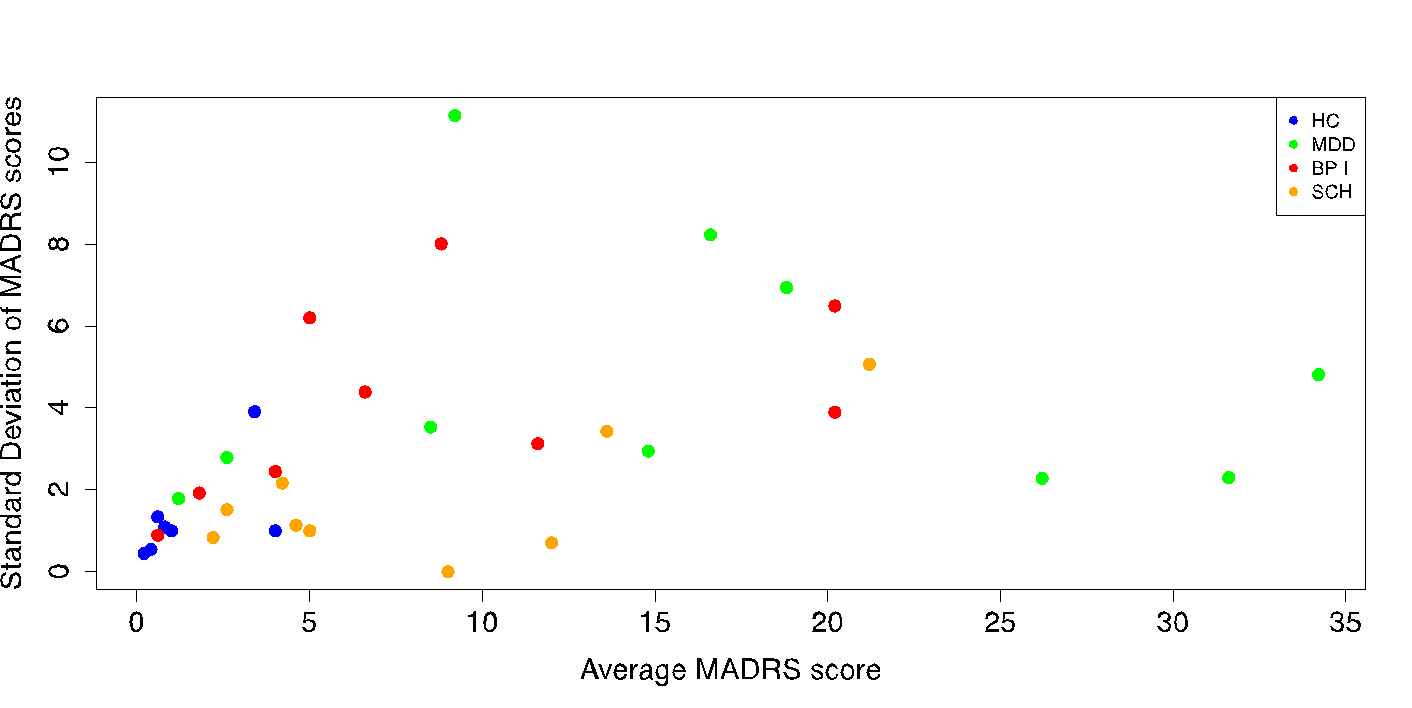
**

**Supplemental Figure 2A-B**. On left (A), completeness of accelerometer and GPS data. The color of the point indicates the phone type, with iOS in red and Android in blue. The dotted diagonal line is a reference line at $y=x$. On right (B), completion rate for each PHQ-8 survey, indicated by the solid black line. The completion rates by diagnostic category are shown using dotted lines. PHQ8 = Patient Health Questionnaire-8; HC = Healthy Control; MDD = Major Depressive Disorder; BP I = Bipolar Disorder; SCH = Schizophrenia/Schizoaffective Disorder.

1. **B.**

**
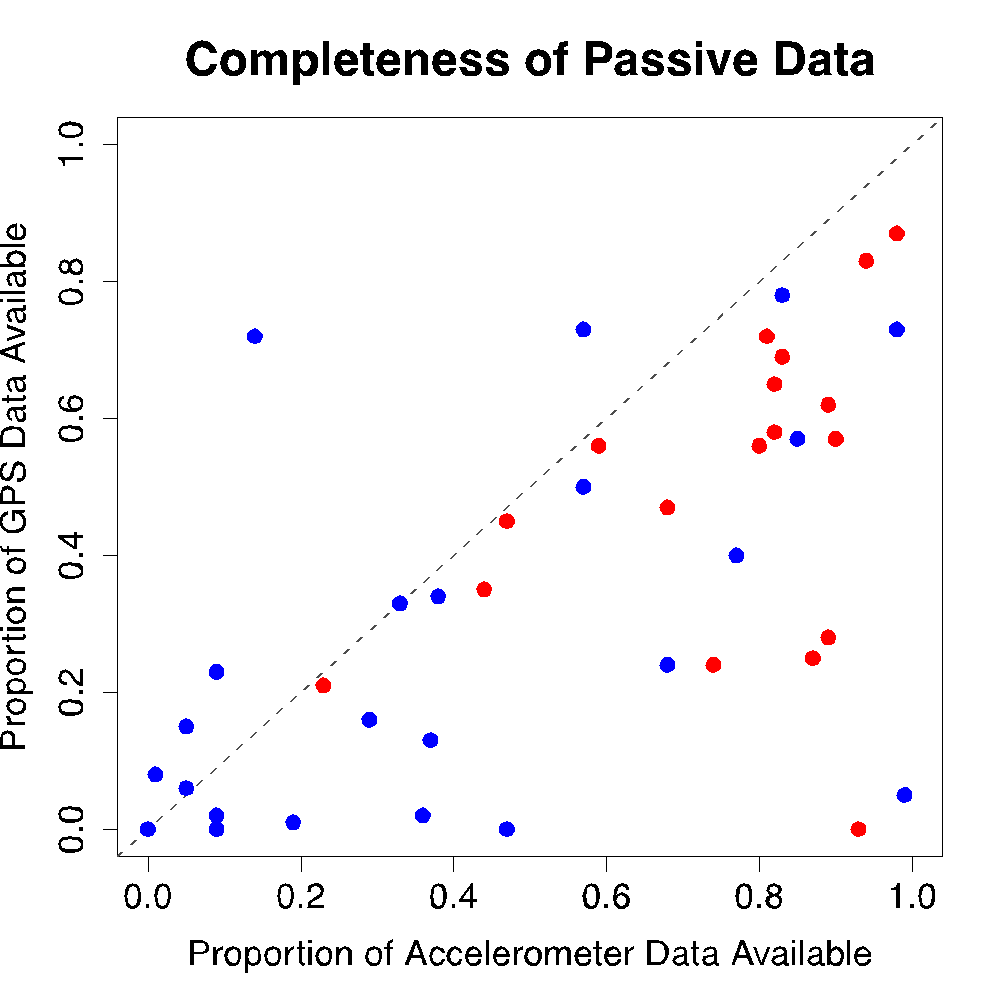
**
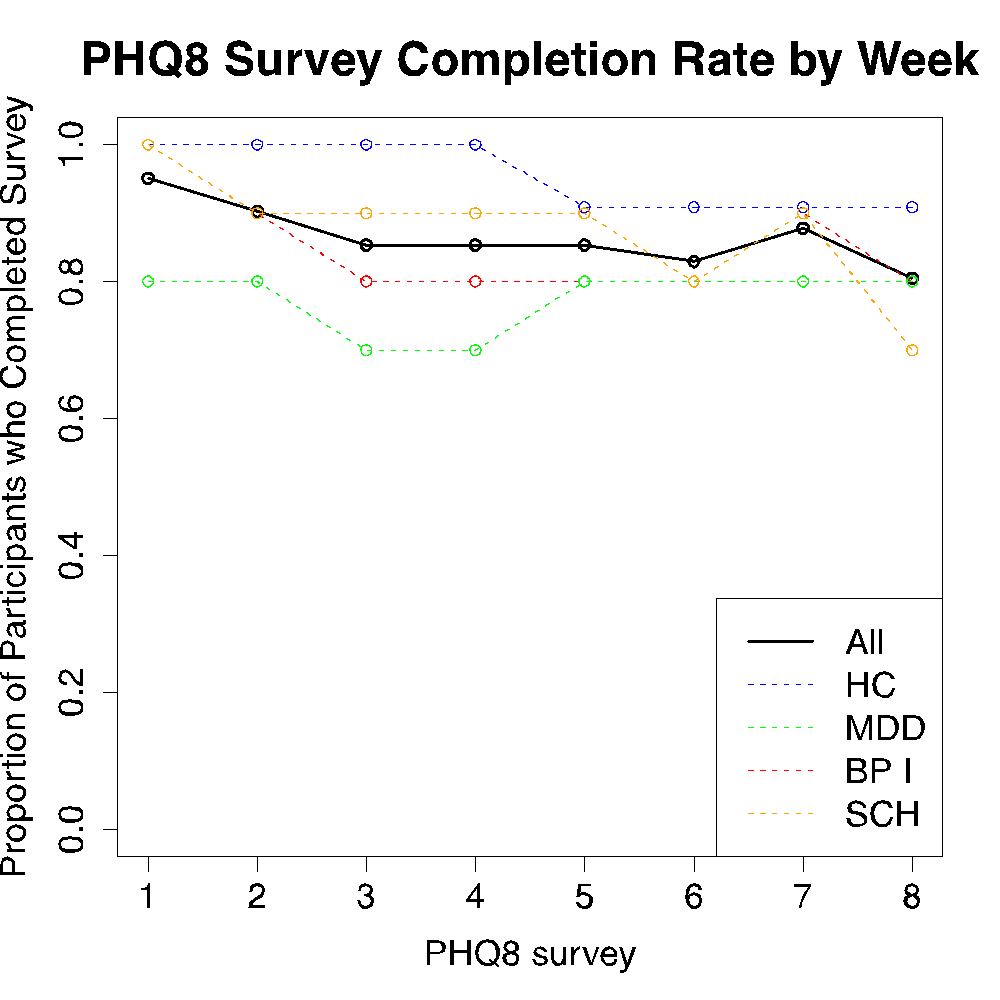


**Supplemental Figure 3.** Histogram of the number of weeks that the participant completed one or more Patient Health Questionnaire (PHQ)-8 surveys.

**
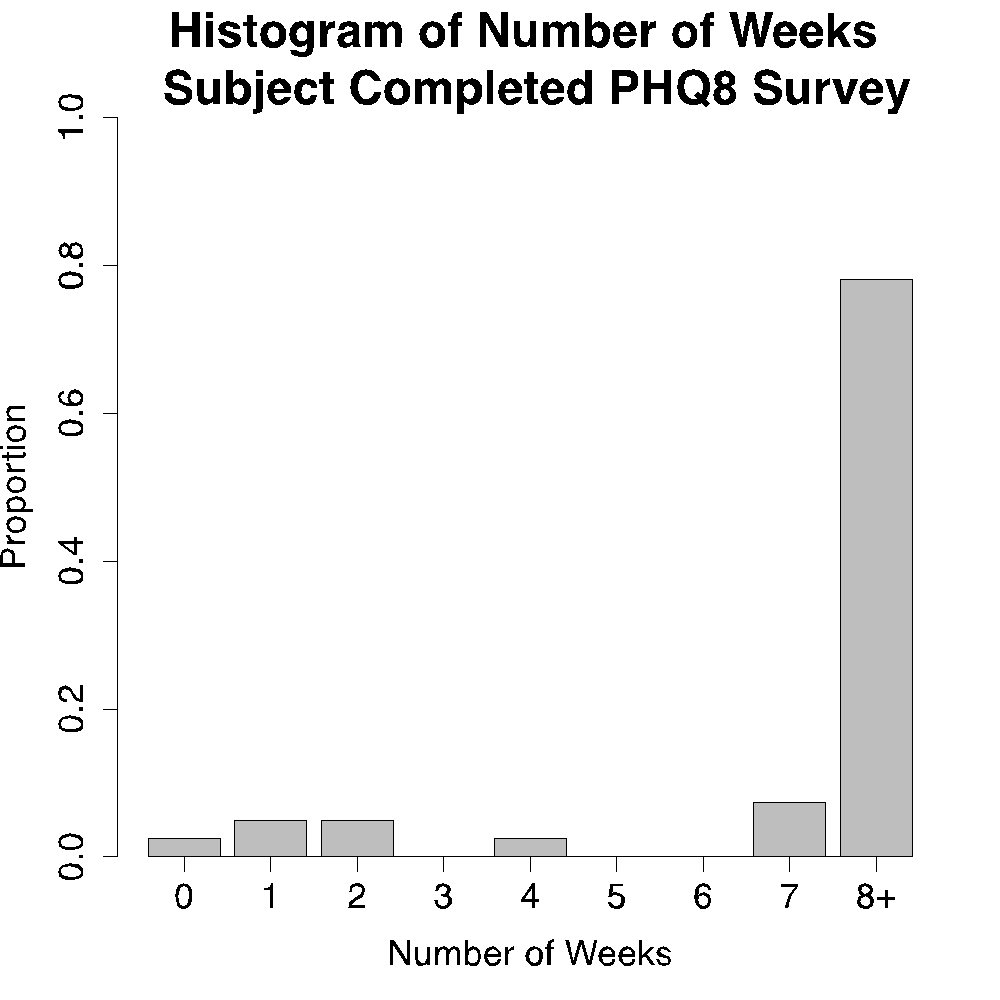
**

**Supplemental Figure 4.** Examples of accelerometer data quality for three participants over the 8-week study period. A shading of white corresponds to no data collected during the hour and black corresponds to data collected at every minute of the hour. The darker the shading, the better the data quality. For each participant’s plot, the *x*-axis shows the week of the follow-up, ranging from the week of the baseline visit to the week of the fourth follow-up visit. The *y*-axis is day of week, with the tick marks occurring at 12:00 AM.


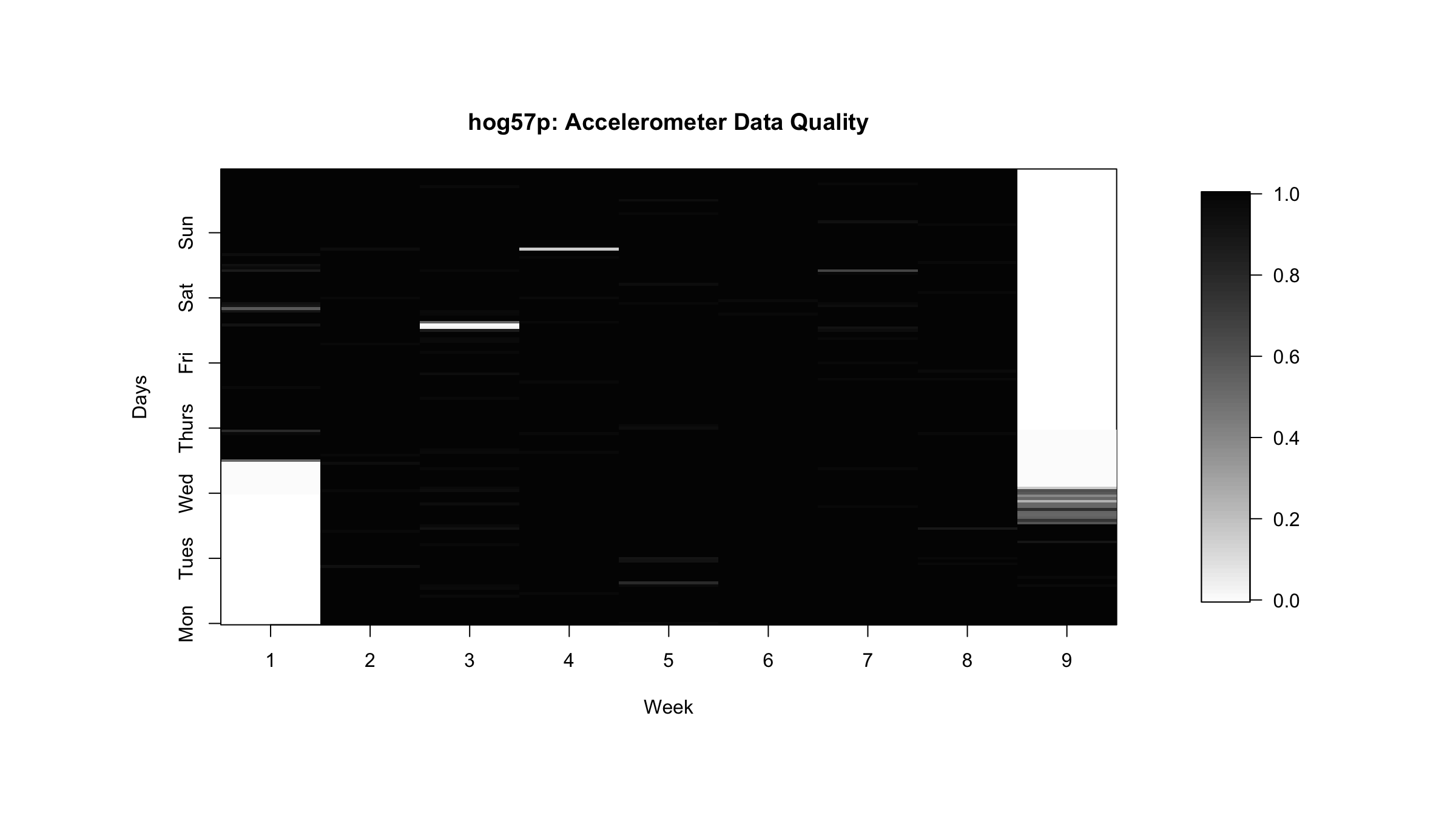

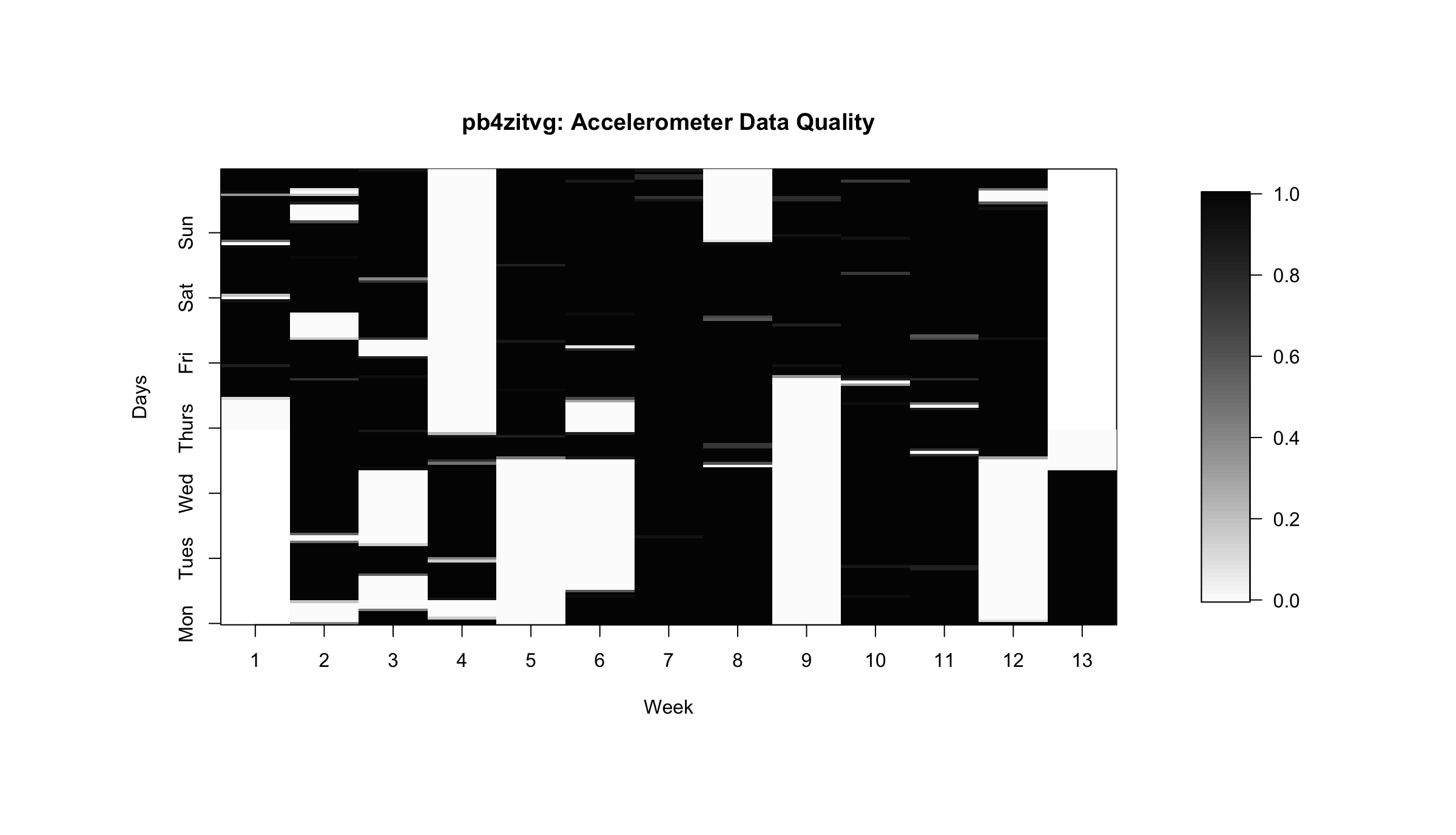

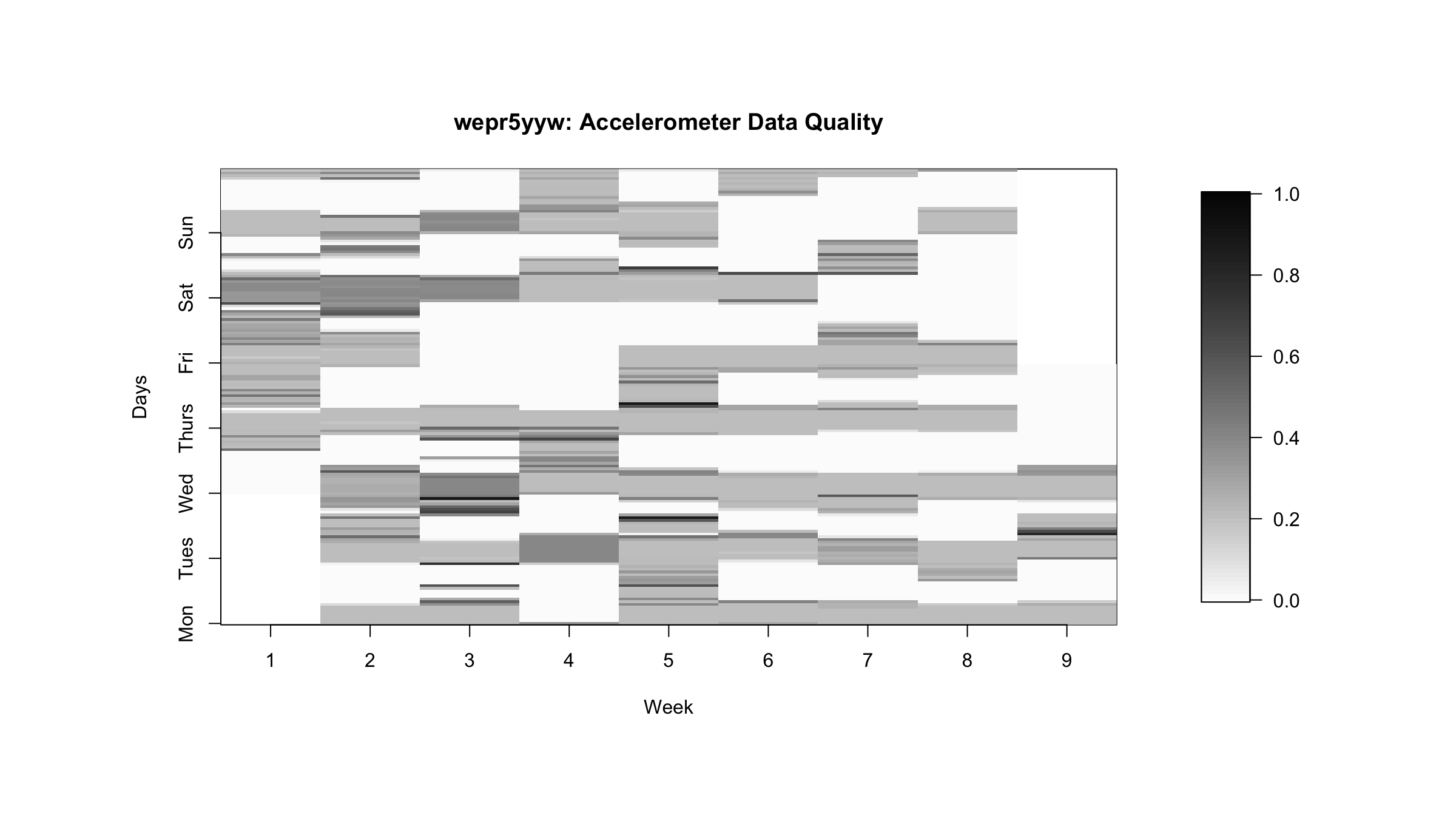

Supplement: Supplementary file 1 — Supplementary Material [file BRB3-12-e02077-s001.docx]
